# Voronoi-Based Heuristic Algorithms for Speed Camera Location Considering Area-Wide Influence

**DOI:** 10.3390/s26217039

**Published:** 2026-11-07

**Authors:** Sebastian Fuenzalida de Regil, Wilfredo Yushimito, Sebastian Moreno

**Affiliations:** Facultad de Ingeniería y Ciencias, Universidad Adolfo Ibáñez, 750 Padre Hurtado Ave., Viña del Mar, Valparaíso 2520000, Chile; sebasfuenzalida40@gmail.com (S.F.d.R.); sebastian.moreno@uai.cl (S.M.)

**Keywords:** speed cameras, spillover effect, location, Voronoi diagrams, Genetic Algorithm, Nelder–Mead

## Abstract

Speed cameras are typically placed at high-crash locations to deter speeding. However, their deterrent effect extends beyond the installation site, as drivers moderate their speed in surrounding areas through halo and spillover effects. This study formulates speed camera location as a continuous facility location problem in which each camera’s influence decays with distance, approximating these effects as a radial, area-wide influence. Voronoi diagrams partition the study area into camera influence regions, and the objective is to maximize the average modeled probability of speed reduction under three assumed distance-decay functions (linear, concave, and convex) with influence distances of 600 to 1000 m. A Nelder–Mead algorithm (NM) and a Genetic Algorithm (GA) are used to relocate a fixed number of cameras, starting from recurrent crash hotspots in Santiago and Providencia, Chile. Results are averaged over 30 independent runs and compared with a greedy relocation heuristic. Relative to the hotspot-based placement, the GA increases the average modeled probability of speed reduction by up to 14.9 percentage points on average and by up to 24.2, outperforming NM and the greedy algorithm. Because the decay functions are assumed rather than calibrated, these gains reflect improved modeled coverage, and empirical validation is needed before safety benefits can be claimed.

## 1. Introduction

Traffic accidents have been recognized as a major global health problem, contributing to a considerable number of injuries and deaths each year. Approximately 1.25 million people die annually worldwide due to traffic accidents, and between 20 and 50 million suffer non-fatal injuries [1,2,3]. Although traffic accidents have multiple factors, including driver behavior, road infrastructure, and vehicle conditions [1,2], excessive or inappropriate speed remains a major contributor to crash risk and injury severity, which increase both the risk of an accident and the severity of the resulting injuries [4,5,6,7].

The World Health Organization (WHO) estimates that reducing the average traffic speed by 5% could decrease the number of fatal traffic accidents associated with speeding by 30%. Likewise, a 10% reduction in average speed translates into a 40% decrease in fatalities in rural areas and slightly less in urban areas [8]. Thus, it seems evident that reducing speed is imperative to mitigate traffic risk.

Speed cameras are among the most effective and widely used tools for reducing vehicle speeds [5,7]. Speed cameras capture an image of vehicles passing by at a predetermined activation speed. The vehicle’s registration data are read from this photographic evidence, enabling authorities to contact and fine the owner. These cameras reduce vehicle speeds by the deterrent effect of fines or other sanctions, such as revocation of the driving license or, in severe cases, imprisonment [9]. The literature indicates that the implementation of this type of speed control and enforcement can reduce traffic accidents by 0% to 55% in the areas where they are deployed [4,5,10,11,12,13,14]. However, the reported extent of a camera’s influence varies widely, from about 200 m to 2 km, depending on whether it is measured through changes in speed or in crashes, and on factors such as the randomness of camera placement and drivers’ ability to identify “safe” and “unsafe” detection zones [11,15].

This is related to the halo effect, a key psychological phenomenon influencing drivers’ behavior around speed cameras. This occurs when drivers, perceiving a heightened enforcement presence, adjust their speed not only at the camera’s location but also in surrounding areas where no actual monitoring occurs. For instance, if a speed camera is installed on a particular street, drivers may assume that nearby roads are also monitored, leading to a broader reduction in speeding. A related but distinct phenomenon is the spillover effect, which refers to how changes in one area—such as installing a speed camera—cause measurable shifts in traffic flow and driver behavior in adjacent areas. While the halo effect is primarily psychological, the spillover effect is often observed in traffic patterns, influencing congestion, rerouting, and speed variations beyond the enforcement zone.

This study addresses the placement of speed cameras to maximize their modeled influence on speed reduction. Rather than modeling the behavioral mechanisms behind the halo and spillover effects, we approximate their combined outcome as a radial, area-wide influence that decays with distance from each camera. This assumption reflects that drivers increasingly know where cameras are, for instance through navigation applications, but not which direction they face or which roads they monitor. In particular, we evaluate two optimization heuristics: the Nelder–Mead (NM) algorithm and a Genetic Algorithm (GA). Both methods incorporate Voronoi diagrams to define the cameras’ areas of influence and consider three types of probability functions (linear, concave, and convex) to model the probability of speed reduction. Their performance is compared against a greedy relocation heuristic used as a benchmark. The methods are evaluated using real-world accident hotspot data from the communes of Santiago and Providencia in the Santiago Metropolitan Region, Chile.

The remainder of this paper is organized as follows. Section 2 reviews the relevant literature, Section 3 defines the problem, Section 4 describes the methodology, Section 5 presents the case study and experimental design, Section 6 discusses the results, and Section 7 concludes the paper.

## 2. Literature Review

Extensive research has been conducted on the impact of speed cameras such as their effects on speed reduction and accident prevention. Most studies rely on before-and-after analyses to evaluate their impact on traffic safety. These studies consistently show that static and section speed cameras significantly improve traffic safety by reducing crashes, injuries, fatalities, and average speeds, while enhancing speed compliance. These effects have been observed across diverse contexts and analytical approaches, including Empirical Bayes, Bayesian, and time series methods, confirming the robustness of their impact in multiple countries such as Norway, Italy, Belgium, France, Rwanda, Qatar, Sweden, and the United States of America [13,14,16,17,18,19,20,21,22,23,24].

Moreover, installed speed cameras can reduce both traffic accidents and average vehicle speeds within their zones of influence, which range from a few hundred meters to several kilometers [25,26]. Additionally, a higher number of cameras has been associated with greater reductions in accidents [27]. The extent and impact of the cameras’ zones of influence, as well as the literature on the locations of speed cameras, are further elaborated in this section.

### 2.1. Halo Effects and Kangaroo Jumps

The zone of influence of a speed camera refers to the area beyond its immediate enforcement point where driver behavior is still affected. As mentioned in the previous section, studies suggest that speed reductions and improved compliance can extend up to several kilometers beyond the camera location, influencing overall traffic flow and safety.

A key phenomenon associated with this extended impact is the halo effect, which describes how drivers tend to maintain lower speeds even after passing a speed camera, either due to continued caution or uncertainty about additional enforcement. This effect can be presented in two different variants: distance halo and the spillover effect. Both effects describe how the influence of a speed camera extends beyond its immediate enforcement location, but they refer to different mechanisms of impact.

The distance halo refers to the gradual decrease in a speed camera’s influence as drivers move away from it [28]. When approaching a camera, drivers slow down to avoid detection; after passing it, some maintain reduced speeds for a certain distance before returning to their usual behavior. The length of this zone depends on road design, driver awareness, and enforcement policies. Most studies report that it extends between 150 and 300 m from the camera, both upstream and downstream, and occasionally up to 500 m [3,10,26]. Using vehicle trajectory data, ref. [26] found that the reduction in average speed can extend up to 1000 m downstream of the camera, approaching zero at that distance. Larger zones of influence, of up to several kilometers, have been reported for area-wide reductions in crashes [25,26]; in this study, we focus on the effect on speed and therefore adopt 1000 m as the extent of a camera’s influence (Section 4.1).

The spillover effect occurs when a speed camera influences driver behavior not only on the monitored road but also on surrounding roads [28]. For example, if a camera is installed on a main road, drivers may also reduce their speed on nearby streets, assuming these might be monitored as well. This suggests that enforcement at one location can improve safety beyond the monitored road, for example, by reducing crashes [29]. However, spillover can also be negative: ref. [28] reviews studies reporting speed reductions of 20% to 67% and crash reductions of 3% to 29% in some areas, but also crash increases in nearby areas when drivers divert to untreated roads. Spillover has also been studied for other speed management measures; for instance, [30] analyzed data from three U.S. corridors (I-85 in Georgia, I-75 in Michigan, and I-84 in Oregon) and found that raising posted speed limits on interstates can affect speeds on adjacent roads, although the evidence was not conclusive. Because spillover operates through the road network and depends on route choice, it cannot be fully captured by a distance-based model. In this study, we approximate the combined halo and spillover effects as a radial influence around each camera (Section 3.1), and we discuss the limitations of this approximation, including diversion to untreated roads, in Section 7.

Similar to halo effects but with opposite consequences, the phenomenon of Kangaroo jumps is also discussed in the literature (see for instance, ref. [28] and references therein). This term refers to the behavior of drivers who reduce their excessive speed abruptly when entering the camera’s influence zone to avoid detection and then resume their excessive speed immediately after leaving the zone [31,32]. While this phenomenon has not been observed to negatively impact safety, it is noteworthy when operating a speed camera system [25]. Since the probability functions used in this study describe the average response of drivers, they do not capture this abrupt, individual-level behavior.

### 2.2. Location of Speed Cameras

Although extensive research has examined the effects of installed speed cameras, relatively few studies have investigated methodologies, criteria, or guidelines for determining optimal locations to maximize the effectiveness of speed cameras. For instance, one of the most commonly mentioned criteria is placing cameras in areas with a historically high incidence of accidents [16,33,34]. Ref. [16] describes the location criterion as areas with above-average numbers of injury crashes or a crash cost of 30% higher than the average. Similarly, ref. [33] agrees with placing cameras in locations with above-average accident numbers related to excessive speed. Other types of models explore the use of indices to select the location of the cameras. For instance, ref. [35] explores an optimal camera location methodology based on two ranking methods: the Crash Index (CI) and the Violation Rate (VR, which uses speed and road condition data to predict speeding drivers’ proportion through regression models). Then, the priority index is calculated as PI=CI×VR. Ref. [7] addresses optimal location using historical crash data, adding a priority score based on the reduction in social costs from camera installation. They start by defining a Segment Score (SS) criterion to highlight road segments with high numbers of severe speed-related accidents. Then, they prioritize zones based on the expected reduction in social costs, iteratively covering all marked zones according to SS. Ref. [36] combines spatial and statistical analysis of infrastructure risks, accident frequency, and severity. A key advantage of this approach is its applicability in regions with limited accident data. They georeference accident data, generate heatmaps, and use kernel density analysis to highlight road sections with a high concentration of crashes, choosing them as candidate locations for speed cameras.

The study [34] mentions that new locations should not have existing speed control measures to accurately measure the cameras’ effectiveness. This criterion is not demonstrated as optimal in the literature, but it is mentioned as the most common strategy used in speed camera programs. Other criteria include placing them on main avenues, loop roads (also known as ring roads, which circle a city or area), or radial roads (where main avenues radiate out from the city center) [37]. Such criteria are common and practical strategies due to the high visibility and constant traffic flow in those areas. However, they are not necessarily considered optimal from a purely effectiveness-based perspective regarding accident reduction and speed control, but rather as a pragmatic solution based on accessibility and frequent traffic.

In light of these considerations, there has been a growing interest in optimization models to enhance the selection of camera locations. For example, ref. [38] proposes an optimization model utilizing machine learning techniques, leveraging historical crash data and road characteristics to determine the most effective camera locations. Essentially, the problem involves using machine learning algorithms to classify roads according to their importance or risk level. In doing so, they develop a supervised classifier that categorizes road segments’ contribution to road safety as good, neutral, or bad, based on a priority index of speed limit violations and speed-related traffic accidents. Good sites correspond to locations where speed cameras are most effective. Among all machine learning models used for classification, the best results were obtained by the Random Forest classifier algorithm. Recently, ref. [39] proposes a causal machine learning approach to determine optimal locations for speed cameras by estimating the specific crash reduction effect at each site. Using UK data, this method prevents more crashes than traditional rules based solely on historical crash frequency, although the latter remains useful when additional information is unavailable.

Using another approach, ref. [40] proposes a genetic algorithm for pattern search to optimize speed camera locations. Using a negative binomial model enhanced by an empirical Bayes method, they predict traffic accidents on various roads and apply the Facility Location Problem [41,42,43] model to determine the optimal number and placement of speed control devices. Then, pattern search and a genetic algorithm are used to evaluate alternative camera locations, looking for a better configuration.

## 3. Problem Definition

The placement of speed cameras is typically at the discretion of authorities, often without specific objectives [36]. For example, in Chile, areas with high accident rates in both urban and rural sectors are usually recommended for camera placement [44]. As explained in Section 2.2, most existing approaches rely on similar placement criteria, while relatively few employ formal location optimization models.

In the literature, two types of location problems are commonly referenced: discrete and continuous. Discrete location problems assume that facilities can be placed at nodes or links in a network, while continuous location problems allow facilities to be placed at any point within a plane [42].

For this research, the speed camera location problem is formulated as a continuous location problem in which each camera has an area-wide zone of influence that includes surrounding roads. This formulation rests on the following assumption. Navigation applications such as Waze or Google Maps alert drivers to the presence of nearby speed cameras and can warn them when they exceed the speed limit; for example, ref. [45] reports that Waze warnings reduce the share of speeding drivers from 23.4% to 19.8%. Drivers can therefore know that a camera is nearby, but generally not which direction it faces or which road it monitors. As a result, they may moderate their speed on any nearby road, not only on the monitored one, which is consistent with the halo (distance) and spillover effects described in Section 2.1. We approximate the combined outcome of these effects as a radial influence that decreases with Euclidean distance from the camera, regardless of road connectivity or camera orientation. This is a simplifying assumption about the aggregate response of drivers rather than a model of the underlying behavioral mechanisms, and its limitations are discussed in Section 7. Additionally, we assume that the speed cameras (in this case, static) can be installed at any point within the study area, not only at predefined finite locations. Section 6.2 assesses the robustness of the results when cameras are restricted to the roadside.

This setting resembles the continuous space p-Median problem [46]. This model aims to locate *p* facilities within a continuous space. Unlike discrete space location problems, where potential locations and clients are predefined discretely, in continuous space problems, locations and clients can take any value within a continuous space. The objective is to minimize the total weighted distance of users to their nearest facility [42]. The next section outlines the general definition of the continuous space p-Median problem, followed by a specific application to the problem of locating speed cameras.

### 3.1. The Continuous Space p-Median Problem

One of the most fundamental continuous location problems is the continuous space p-Median problem (CSPMP) [47,48]. This problem involves finding a set of locations for *p* facilities to minimize the total weighted distance of users to their nearest facility. Ref. [48] formally defines the CSPMP as follows: consider a set of *p* facilities providing the same service in a region *S*, with locations $x_{1}$ to $x_{p}$. The demand for services from users is continuously distributed within *S*, with demand density at a point *x* equal to ϕ(x). Users go to the nearest facility among the *p* available facilities, and the access cost for a user is expressed as a function f(t) of the distance *t* between the user and their nearest facility. Under these conditions, the problem is to find the locations of the *p* facilities that minimize the total access cost for the users. Let $X=\{x_{1},\ldots ,x_{p}\}$, the objective function, which represents the total access cost for all users, is

(1) $$F\left(X\right)=F\left(x_{1},\ldots ,x_{p}\right)=\int_{S}f\;\left(\min_{i}∥x-x_{i}∥\right)\phi \left(x\right)\;dx$$

where ∥·∥ denotes the Euclidean norm, so that $t=\min_{i}∥x-x_{i}∥$ is the distance from point *x* to its nearest facility, and f(t) is a non-decreasing function that converts this distance into an access cost. A common choice is $f\left(t\right)=t^{2}$, the squared Euclidean distance, for which the optimal facility locations are the centroids of their Voronoi regions [49]. Minimizing F(X) is an unconstrained nonlinear optimization problem with 2p variables. As [49] shows, Equation (1) can be reformulated in terms of the Voronoi diagram, as explained in Section 3.3.

### 3.2. Speed Camera Localization Problem

We proceed to adapt the model formulation in Equation (1) to the speed camera location problem. Similar to the problem stated in Section 3.1, we consider a set of *p* speed cameras to be placed in a region *S* with drivers continuously distributed within *S*. Rather than an access cost that increases with distance, we consider that the probability that a driver reduces speed decreases with the distance *d* to the camera, following a function pr(d). The objective is therefore to maximize the overall probability that drivers reduce their speed:

(2) $$G\left(X\right)=G\left(x_{1},\ldots ,x_{p}\right)=\int_{S}pr\;\left(\min_{i}∥x-x_{i}∥\right)\phi \left(x\right)\;dx,$$

where:

- pr(d) is the probability of speed reduction at distance *d* from the nearest camera, a non-increasing function with pr(0)=1 and pr(d)=0 for $d\geq d_{\max}$ (Section 4.1);
- $x_{i}$ is the location of camera *i*;
- ϕ(x) is the density of drivers at point *x*. In this study, ϕ(x) is uniform over *S*, since data on the spatial distribution of drivers were not available.

To compare configurations across study areas, we report the spatial mean

(3) $$\bar{G}\left(X\right)=\frac{G(X)}{\int_{S}\phi \left(x\right)\;dx},$$

which can be interpreted as the average probability of speed reduction over *S*. Because pr(·) decreases with distance, dispersed configurations that leave few points far from every camera are favored, whereas clustered configurations are penalized. $\bar{G}\left(X\right)$ takes values between 0 and 1 and is reported as a percentage throughout the paper. Accordingly, differences between configurations are expressed in percentage points.

### 3.3. Voronoi Approach to the Speed Camera Localization Problem

One method used to solve continuous location problems considers the use of Voronoi diagrams [47,48,49]. Given a set of seed points, or sites, a Voronoi diagram divides the plane into convex polygons, where each polygon contains all points that are closer to its seed than to any other seed. The edges of the polygons are segments of the perpendicular bisectors between neighboring seeds. Efficient computational methods for constructing Voronoi diagrams have enabled the development of algorithms for this type of problem [42].

Since pr(·) is non-increasing in distance, the nearest camera yields the highest probability at every point, i.e., $\max_{i}pr\left(∥x-x_{i}∥\right)=pr\left(\min_{i}∥x-x_{i}∥\right)$. The Voronoi regions $V_{i}$ of the cameras therefore partition *S* into the areas served by each camera [50], and the objective in Equation (2) can be decomposed exactly as

(4) $$G\left(X\right)=\sum_{i=1}^{p}G_{i}=\sum_{i=1}^{p}\int_{V_{i}}pr\;\left(∥x-x_{i}∥\right)\phi \left(x\right)\;dx,$$

where $G_{i}$ is the contribution of camera *i* to the speed reduction of the drivers in its Voronoi region $V_{i}$. Because squaring is monotonic, this partition is the same whether Euclidean or squared Euclidean distance is used. In the Voronoi diagram, the cells of the cameras located near the boundary of the study area are unbounded. All cells are therefore clipped to the study area *S*, so that $\bar{G}\left(X\right)$ is evaluated only over *S*. This applies to all configurations, including the hotspot-based placement. In practice, each $G_{i}$ is estimated numerically on a regular grid covering the study area, and the resulting objective function G(X) is maximized using the optimization methods described in Section 4.

## 4. Methodology

The overall methodology consists of two steps. First, we generate a set of seed points, which are the locations that will define the Voronoi cells. Each seed point generates a region, and every point in the plane is assigned to the nearest seed point based on Euclidean distance, forming a Voronoi cell around each seed. Second, we optimize the positions of the seed points using an improvement method based on an objective function.

In the case of speed camera location, the seed points are the initial locations of the speed cameras, given by the recurrent crash hotspots (Section 5.1). For a given configuration, a single Voronoi diagram is constructed, and the objective G(X) is evaluated by integrating the probability of speed reduction over each Voronoi region (Equation (4)). The camera locations are then modified iteratively by the Nelder–Mead or Genetic Algorithm operators described in Section 4.2, and the Voronoi diagram and objective are recomputed for each candidate configuration. The process stops when the objective no longer improves or the iteration limit is reached. The next sections describe how the Voronoi diagram is constructed and how the camera locations are improved.

### 4.1. Constructing Voronoi Diagrams for Speed Camera Location

A Voronoi diagram partitions/segments space into regions. Each region corresponds to a site *s* and consists of all the points that are closer to *s* than to any other site [51]. There are several intuitive methods for constructing a Voronoi region for a given point within a set *S*. One consists of taking all the perpendicular bisectors of the segments connecting *s* with the other members of *S*. These bisectors are then used to delimit half-planes. The intersection of all the half-planes containing *s* forms the Voronoi region for *s*. Another method involves starting with the segments connecting *s* to all other points in *S*. Then, the lines are gradually extended outward along the perpendicular bisectors of these segments until they intersect [52].

Based on this idea, a Voronoi Diagram can be applied to the speed camera localization problem. Algorithm 1 details the construction of a Voronoi Diagram for a point s∈S, where *s* is the location of the camera. Each location *s* serves as a seed point and the bisector method [52] is used to construct the Voronoi cells (lines 2–12).
**Algorithm 1** Voronoi Diagram Construction and Probability Estimation  1:Let *X* be a set of locations of speed cameras in a designated area *S*  2:**for** each point s∈X, treat *s* as a seed for the Voronoi diagram **do**  3:      Connect each point *s* with all other points in *X* using straight line segments.  4:**end for**  5:**for** each line segment connecting *s* to other points in *X* **do**  6:      Compute the perpendicular bisector of the segment  7:      Extend the bisector outwards from the segment  8:      **while** bisectors are extending **do**  9:            Continue extending bisectors until they intersect with other bisectors10:            Mark the intersection as a vertex of the Voronoi diagram11:      **end while**12:**end for**13:**for** each Voronoi cell corresponding to a point s∈X **do**14:      Calculate the probability of drivers slowing down inside the Voronoi cell15:**end for**16:**return** the Voronoi diagram and the probabilities of slowing down for each cell

**Figure 1:**
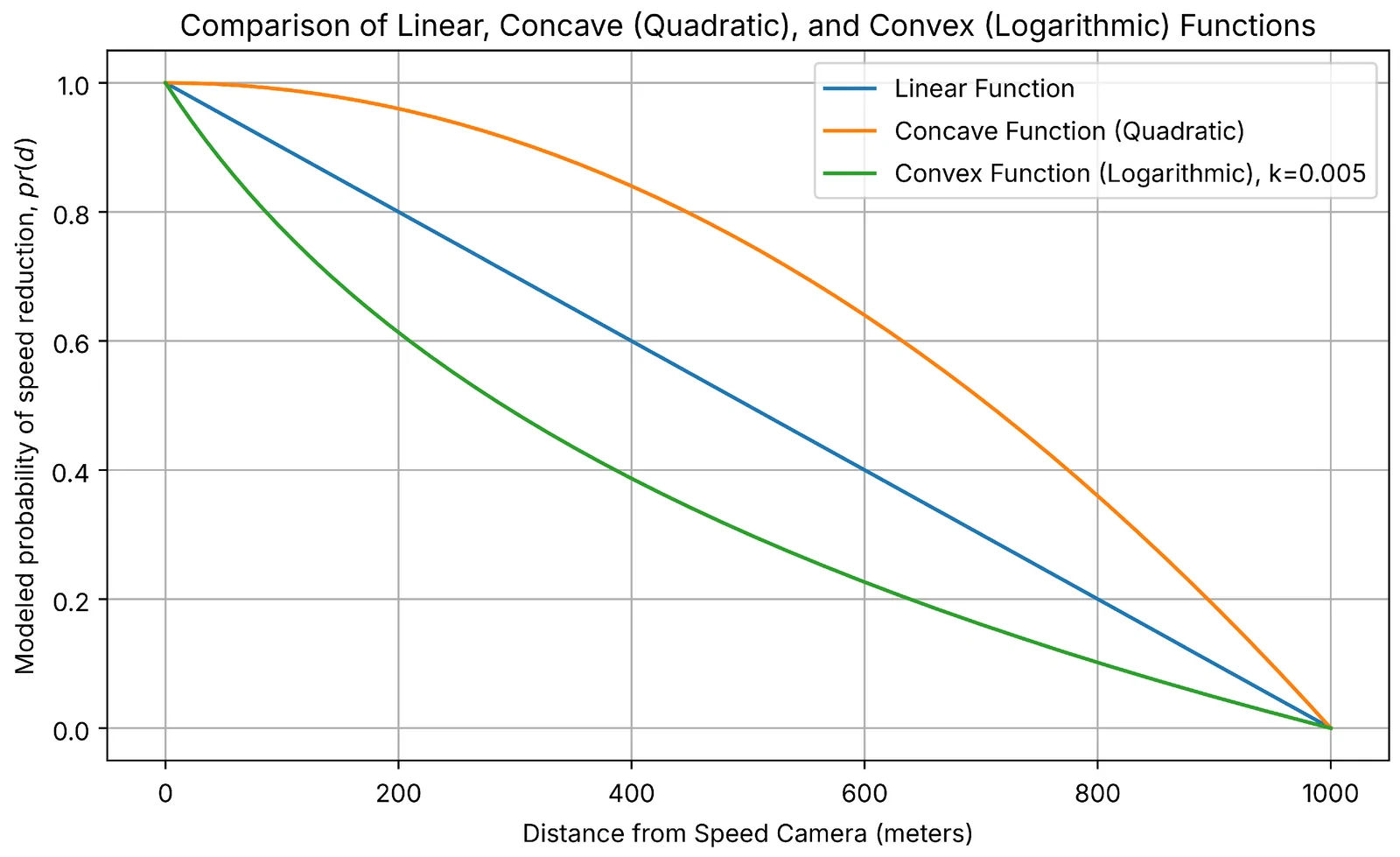
Assumed speed-reduction functions pr(d) as a function of the distance to the camera: linear, concave (quadratic), and convex (logarithmic). All functions equal 1 at the camera location and 0 at distances of 1000 m or more ($d_{max}=1000$).

After the Voronoi diagram is constructed, the contribution of each camera is computed by integrating a function pr(d) that describes how the influence of the camera on speed decreases with the Euclidean distance *d* (in meters) to its location. Using empirical data, [26] found that the reduction in average speed is largest at the camera location and close to zero at a distance of 1000 m. Based on this evidence, we define pr(d) as a normalized influence function, equal to 1 at the camera location and to 0 at distances of 1000 m or more, which we interpret as the relative probability that a driver reduces speed. Note that [26] supports the extent of the influence rather than these exact values: the reduction in average speed is not equivalent to the probability that an individual driver slows down, and the shape of pr(d) between both endpoints is not known. We therefore consider three alternative functions with different decay behavior, shown in Figure 1: linear (Equation (5)), slow initial decay function or concave (Equation (6)), and fast initial decay function or convex (Equation (7)). Any of these functions can be used to estimate the probability of speed reduction within each Voronoi cell (lines 13–15 in Algorithm 1). Calibrating pr(d) with individual speed data is left for future work (Section 7).

(5) $$\mathrm{Linear}\;\mathrm{Function}:\;pr\left(d\right)=-\frac{1}{d_{max}}d+1$$

(6) $$\mathrm{Concave}\;\mathrm{Function}\;(\mathrm{Quadratic}):\;pr\left(d\right)=-\frac{1}{d_{max}^{2}}d^{2}+1$$

(7) $$\mathrm{Convex}\;\mathrm{Function}\;(\mathrm{Logarithmic}):\;pr\left(d\right)=1-\frac{\log(1+0.005d)}{\log(1+0.005\cdot d_{max})}$$

Equations (5)–(7) apply for $0\leq d<d_{max}$, and pr(d)=0 for $d\geq d_{max}$, where $d_{\max}$ is the maximum distance of influence of a camera. In the base setting, $d_{\max}=1000$ m, the distance at which [26] found the reduction in average speed to be close to zero. Also, all three functions therefore satisfy pr(0)=1 and pr(d)=0 for $d\geq d_{max}$, and differ in how fast the influence decays: the quadratic function decays slowly near the camera, the logarithmic function decays quickly, and the linear function lies in between. Section 5.2 describes how they are adjusted for shorter influence distances in order to assess the sensitivity of the results to this assumption.

### 4.2. Improvement Methods for Speed Camera Location

As discussed in Section 4.1, for a given speed camera location *s*, it is possible to compute the probability of speed reduction within its corresponding Voronoi cell. Since each speed camera location serves as the seed of a Voronoi cell, relocating a camera changes the Voronoi partition and, consequently, the probability of speed reduction across the study area. This property allows us to optimize camera locations using the probability functions described at the end of Section 4.1 as objective functions. In this section, we introduce three iterative methods for improving speed camera placement: the Nelder–Mead (NM) algorithm, a Genetic Algorithm (GA), and a greedy relocation heuristic used as a benchmark. All three methods iteratively adjust camera locations, updating the Voronoi partition and evaluating the resulting probability of speed reduction, with the aim of maximizing the overall objective function until a predefined stopping criterion is met.

#### 4.2.1. Nelder–Mead Algorithm

The Nelder–Mead simplex method [53] is a derivative-free optimization algorithm that iteratively improves the locations of speed cameras. At each iteration, the method maintains a simplex composed of n+1 candidate solutions in an *n*-dimensional search space, where each vertex represents a complete camera configuration. The objective function is evaluated at every simplex vertex to assess the quality of the corresponding solution.

For the speed camera placement problem, the objective function is defined as the negative of the overall probability of speed reduction, G(X), computed using one of the probability functions presented in Equations (5), (6), or (7). Since the Nelder–Mead algorithm performs minimization, the optimization problem is reformulated as

$$\max G\left(X\right)=\min\left[-G(X)\right].$$

Thus, minimizing the objective function is equivalent to maximizing the overall probability that drivers reduce their speed in the presence of speed cameras.

Starting from an initial feasible camera configuration, the algorithm first constructs an initial simplex (Algorithm 2, line 1). Each simplex vertex corresponds to a candidate camera configuration. For every vertex, Algorithm 1 is executed to construct the corresponding Voronoi diagram, estimate the probability of speed reduction within each Voronoi cell, and compute the objective value G(X) (line 3). The simplex is then updated through the standard Nelder–Mead operations of reflection, expansion, contraction, or shrink to generate improved candidate solutions (line 4). A camera configuration is feasible if (i) all cameras lie within the study area *S*, a 5000 m × 5000 m square, and (ii) every pair of cameras is at least $d_{\min}=250$ m apart. Whenever a newly generated solution is infeasible, a repair procedure is applied that preserves the locations of fixed cameras (line 5). The procedure restores fixed cameras to their original coordinates, moves any camera located outside *S* to the nearest point on its boundary, and, for each pair of cameras closer than $d_{\min}$, moves the non-fixed camera (or one of them, if neither is fixed) away from the other along the line joining them until their distance equals $d_{\min}$. These repairs are repeated until the configuration is feasible. The simplex updates and repairs continue until the stopping criterion is satisfied (line 6), after which the algorithm returns the best feasible configuration found, that is, the one with the highest overall probability of speed reduction (line 7). Feasibility does not restrict cameras to roadside locations. However, later in Section 6.2 we evaluate the effect of this simplification.

The convergence criteria were left at SciPy’s default values. Specifically, the default absolute tolerance for changes in the solution vector was $\mathrm{xatol}=10^{-4}$, and the default absolute tolerance for changes in the objective function value was $\mathrm{fatol}=10^{-4}$. The parameter xatol controls the acceptable absolute variation between successive solution vectors. In this study, the solution vector consists of the flattened coordinates of all speed camera locations; therefore, xatol determines whether the candidate camera configurations have effectively stabilized. Similarly, fatol specifies the acceptable absolute variation in the objective function −G(X), and determines whether successive iterations produce negligible improvements in the overall probability of speed reduction. Consequently, the optimization terminates when both convergence tolerances are satisfied or when the maximum iteration limit of 1000 iterations is reached.
**Algorithm 2** Nelder–Mead optimization for speed camera placement**Require:** Initial feasible camera configuration
**Ensure:** Optimized camera configuration
  1:Construct the initial simplex from the initial feasible camera configuration.  2:**repeat**  3:      Evaluate every simplex vertex using Algorithm 1 to compute G(X).  4:      Update the simplex through reflection, expansion, contraction, or shrink operations.  5:      Repair infeasible solutions to satisfy the minimum-distance constraint while preserving fixed camera locations.  6:**until** the convergence tolerances are satisfied or the maximum number of iterations is reached  7:Return the camera configuration with the maximum overall probability of speed reduction.


#### 4.2.2. Genetic Algorithm

Genetic algorithms are adaptive heuristic search algorithms inspired by natural evolution [40]. They are particularly suitable for solving optimization problems that would otherwise be difficult for traditional methods due to complexity, non-linearity, or the large size of the search space [40]. They work by maintaining a population of candidate solutions, called individuals, which evolve over multiple generations. In each generation, individuals are evaluated using a fitness function that measures how well they solve the problem. The best-performing individuals are selected for reproduction through processes like selection, crossover (which combines parts of two parents/“previous solutions”), and mutation (which introduces random variations in a parent). This process gradually improves the population by promoting traits associated with higher fitness. Over time, the algorithm converges toward optimal or near-optimal solutions for complex problems where traditional methods may struggle [54].

For the speed camera placement problem, the population consists of a set of feasible camera configurations, where each individual represents a complete placement of all speed cameras. The fitness function evaluates the quality of each individual by computing the overall probability of speed reduction G(X), using one of the probability functions pr(·) described in Section 4.1. Specifically, for each candidate camera configuration, Algorithm 1 constructs the corresponding Voronoi diagram, estimates the probability of speed reduction within each Voronoi cell, and aggregates these values to obtain G(X).

At each generation, parent solutions are selected through tournament selection, in which a subset of individuals is randomly sampled from the population and the individual with the highest fitness is chosen for reproduction. In addition, an elitist strategy is adopted to preserve the best-performing solutions across generations. Before crossover and mutation are applied, the elite individuals are copied directly into the next generation without modification. This mechanism prevents the loss of high-quality camera configurations and promotes convergence toward increasingly effective solutions.

Algorithm 3 summarizes the optimization procedure adopted in this study. Starting from an initial population of feasible camera configurations, each individual is evaluated by executing Algorithm 1 to compute the fitness value G(X). Parent solutions are then selected using tournament selection, and new offspring are generated through crossover and mutation. In the case of crossover, two parent configurations are combined as a weighted average of the coordinates of corresponding cameras, $x_{i}^{\mathrm{child}}=\alpha \;x_{i}^{A}+\left(1-\alpha \right)\;x_{i}^{B}$, where the weight α is drawn uniformly from [0,1]. In the case of mutation, a randomly selected subset of non-fixed cameras is moved to new random positions. Since these operators may produce infeasible camera configurations (for example, crossover can place cameras closer than $d_{\min}$, and mutation can move a camera outside *S*), the repair procedure described in Section 4.2.1 is subsequently applied to each offspring. Finally, the next generation is formed by combining the elite individuals with the repaired offspring.
**Algorithm 3** Genetic algorithm for speed camera placement**Require:** Initial feasible camera configuration, GA hyperparameters
**Ensure:** Optimized camera configuration
  1:Generate an initial population of feasible camera configurations.  2:Incorporate fixed camera locations into every individual.  3:**repeat**  4:      Evaluate each individual by executing Algorithm 1 to compute G(X).  5:      Select parent solutions using tournament selection.  6:      Copy elite individuals to the next generation.  7:      Generate offspring through crossover and mutation.  8:      Repair offspring to preserve fixed camera locations and satisfy the minimum-distance constraint.  9:      Complete the next generation with the repaired offspring.10:**until** the stopping criterion is satisfied11:**return** the feasible camera configuration with the highest overall probability of speed reduction.


#### 4.2.3. Greedy Relocation Heuristic (Benchmark)

To assess the value of NM and GA beyond the hotspot-based placement, we implemented a greedy relocation heuristic as a benchmark (Algorithm 4). Starting from the hotspot-based configuration (perturbed at the beginning of each run, as described later in Section 5.2), the heuristic relocates one camera at a time, keeping all other cameras fixed. At each iteration, the eligible cameras are those that are not fixed, have not reached a maximum number of relocations, and are located at hotspots with a low number of recorded crashes (below a predefined threshold); among them, the camera with the lowest crash count is selected, so that cameras at the most dangerous sites are relocated last. The selected camera is moved to the candidate position that yields the largest increase in $\bar{G}\left(X\right)$, among positions that satisfy the feasibility conditions of Section 4.2.1. If no candidate improves the objective, the camera is not moved. The procedure stops when no eligible camera can be improved or after 1000 iterations. Unlike NM and GA, which modify all cameras simultaneously, the heuristic changes one camera at a time and accepts only improving moves, so it may stop at a local optimum.
**Algorithm 4** Greedy Relocation Heuristic  1:Initialize camera positions at the recurrent crash hotspots.  2:**for** each iteration up to a maximum of *K* iterations **do**  3:      Construct the Voronoi diagram and compute $\bar{G}\left(X\right)$ (Algorithm 1).  4:      Identify eligible cameras: non-fixed, moved fewer than *M* times, and with fewer than *A* crashes.  5:      Select the eligible camera with the lowest crash count.  6:      Find the feasible position that most increases $\bar{G}\left(X\right)$.  7:      **if** no improving position exists **then**  8:            Mark the camera as non-improvable.  9:      **else**10:            Move the camera to that position.11:      **end if**12:      **if** no eligible camera can be improved **then**13:            **break**14:      **end if**15:**end for**16:**return** the final camera configuration and $\bar{G}\left(X\right)$.

## 5. Case Study and Experimental Design

### 5.1. Data and Base Case Scenarios

For this investigation, we obtained data from the National Commission on Safety (CONASET) [55]. The data consists of available data stating critical transit crash locations from two municipalities (Santiago Centro and Providencia) in the Metropolitan Region of Santiago de Chile, considering a time interval from 2021 to 2022. After data exploration, we observed that critical crash locations repeated over the years with little variation. With this information, the base case was established, assuming that the area of recurrent crashes (critical both years) has a speed camera, as suggested in practice. Note that these locations do not correspond to existing speed cameras; they represent a hotspot-based placement. Each instance is defined on a study area *S* of 5000 m × 5000 m. Finally, we assume that each site provides radial coverage of 360 degrees, either because it is equipped with a 360-degree camera or with four 90-degree wide-angle cameras, or because drivers know where the site is but not which direction it monitors (Section 3.1).

**Figure 2:**
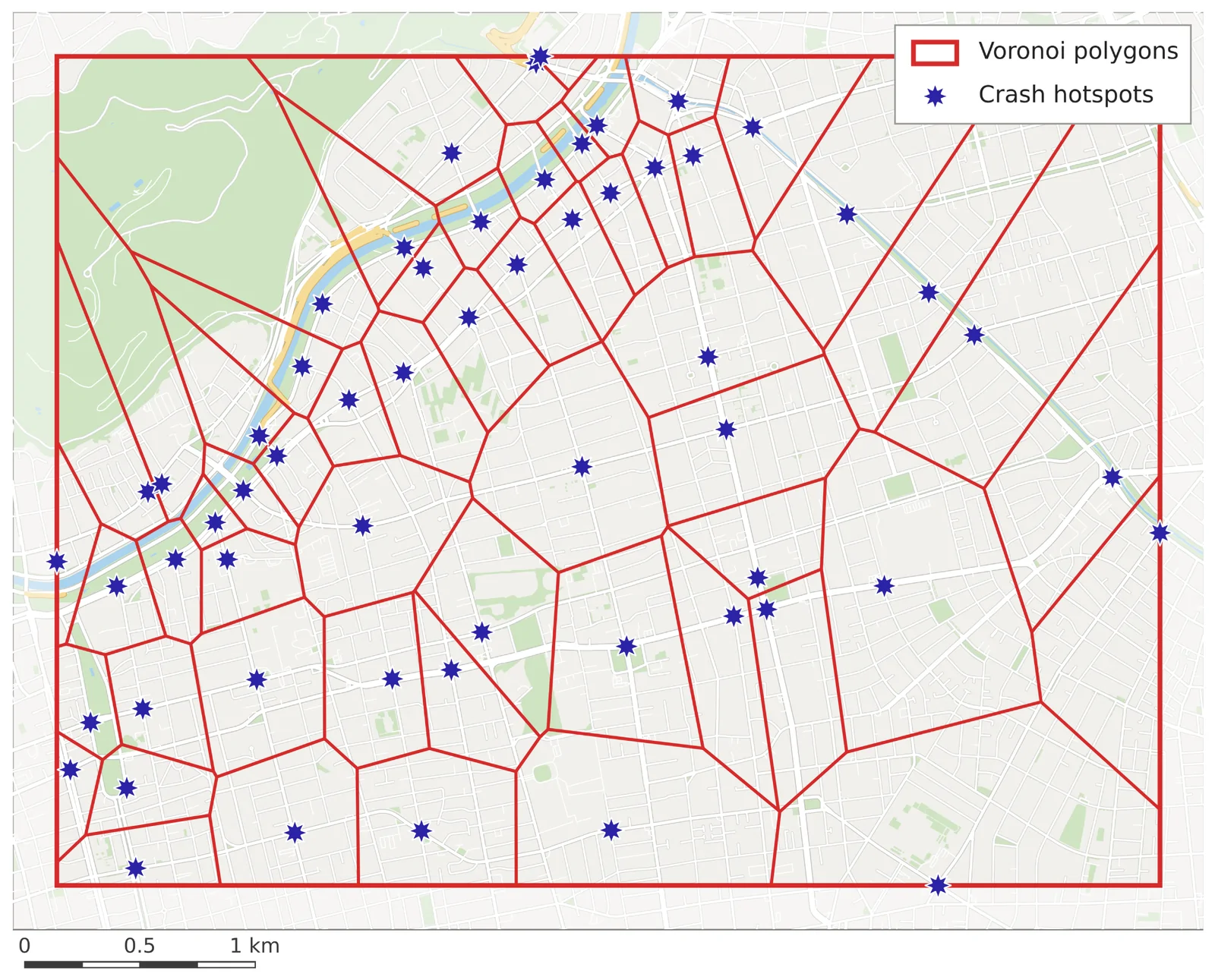
Voronoi Diagram for Providencia.

**Figure 3:**
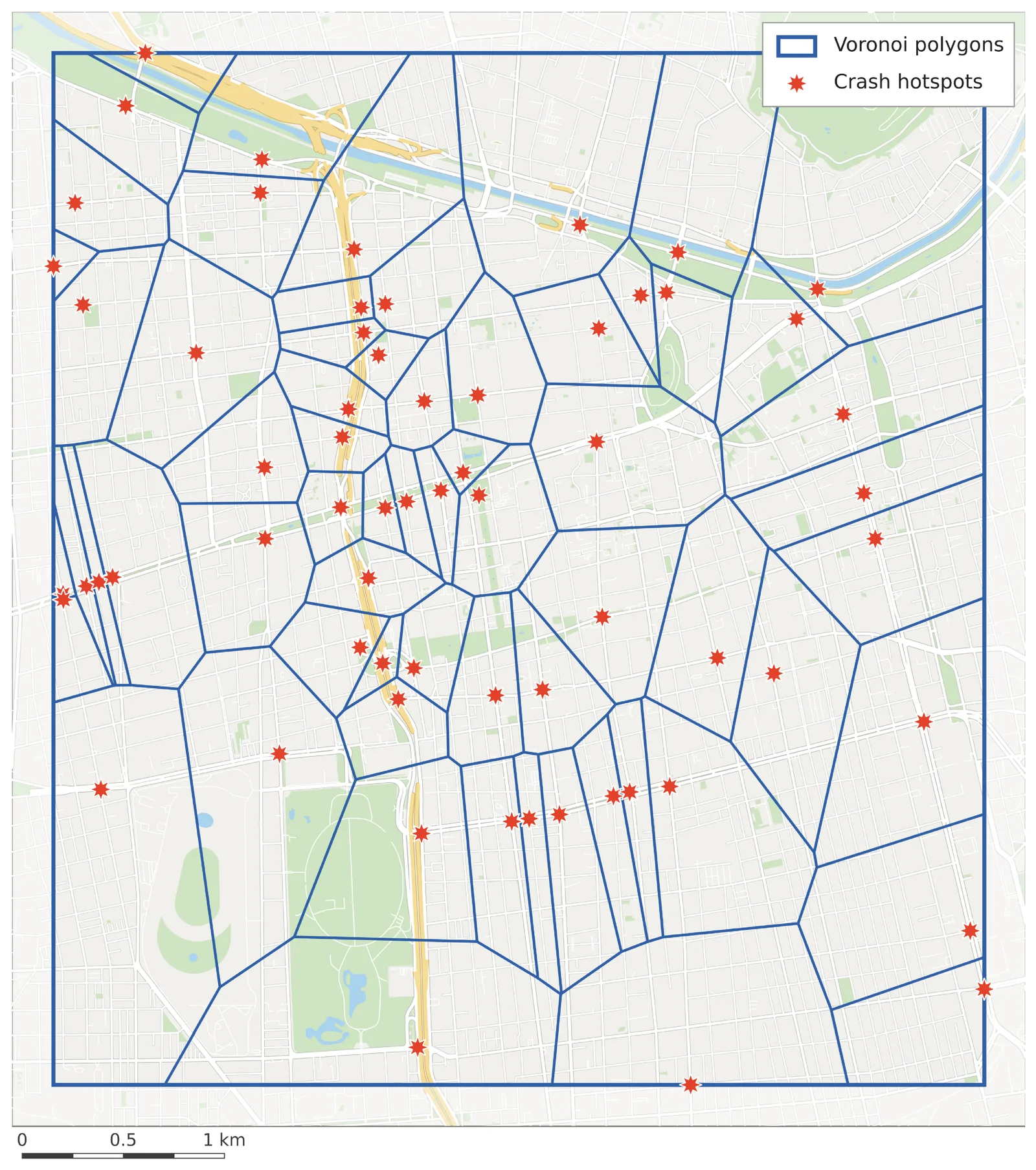
Voronoi Diagram for Santiago.

As a result, we designed two base case instances: Providencia with p=58 cameras (Figure 2) and Santiago Centro with p=65 cameras (Figure 3). The number of cameras equals the number of hotspots, so that the base case and the optimized configurations use the same number of cameras, and it remains fixed during the optimization; only the camera locations change.

**Figure 4:**
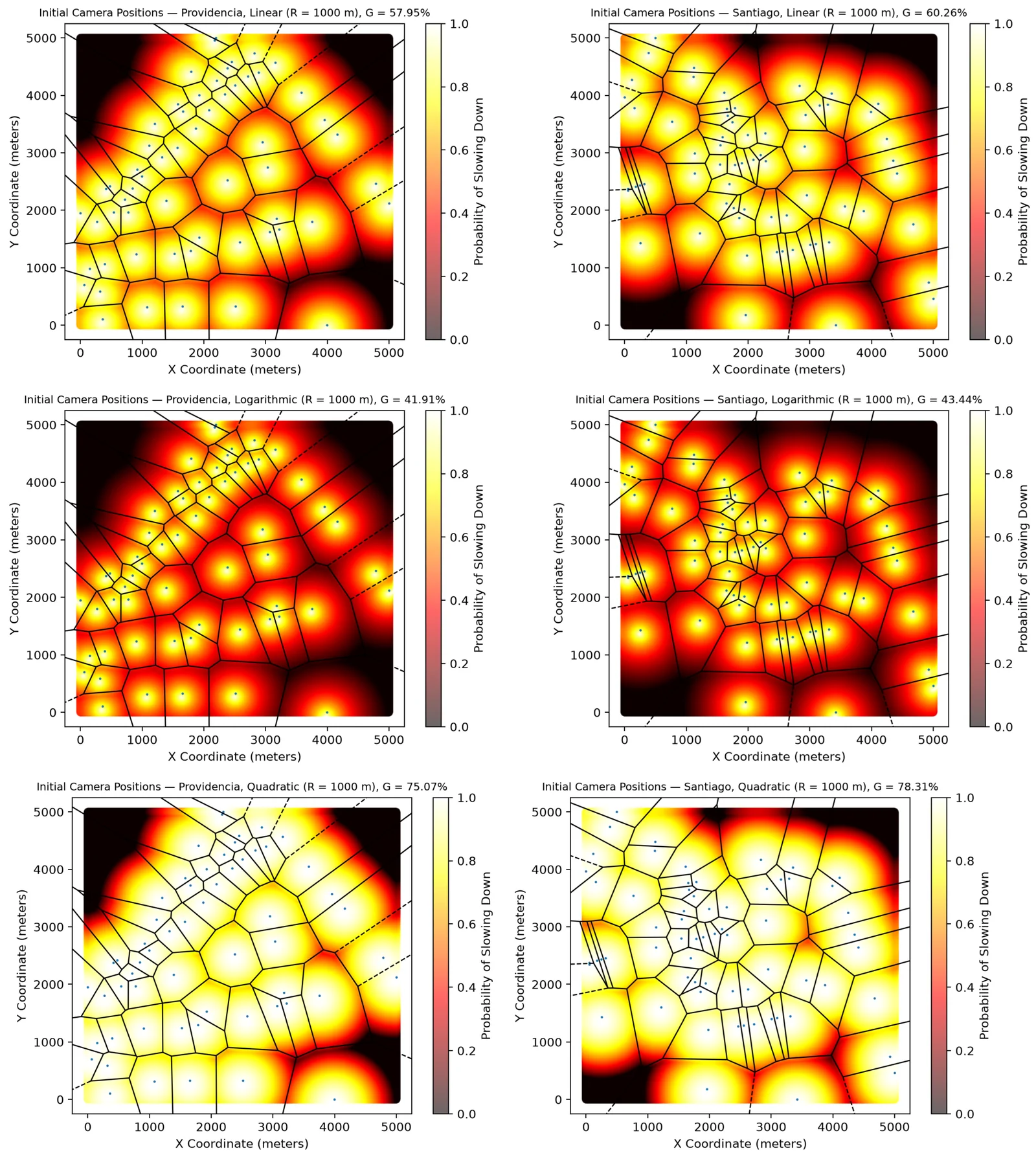
Voronoi Diagrams with the heatmap of the average probability of speed reduction in each Voronoi region for Providencia (**left**) and Santiago (**right**) for linear (**first row**), logarithmic (**second row**), and quadratic (**third row**) functions, for $d_{max}$ = 1000 m.

Then, for each instance, we constructed Voronoi diagrams (following Algorithm 1) using the linear, logarithmic, and quadratic functions considered in Section 4.1, and we obtained the average modeled probability of speed reduction over the study area, $\bar{G}\left(X\right)$ (Equation (3)). As shown in Figure 4, the initial locations are predominantly concentrated along main avenues and intersections. Notably, there are highly problematic areas with numerous points clustered in specific zones or streets, while others exhibit very low coverage.

### 5.2. Experiments

To evaluate the algorithms designed in Section 4, we used the two base case instances described in Section 5.1. That is, both Providencia and Santiago Centro are tested with three proposed probability functions: linear (5), quadratic (6), and logarithmic (7). To assess the sensitivity of the results to the extent of influence, each function was evaluated with $d_{\max}$ = 600, 800, and 1000 m, giving a total of 18 experimental settings (2 instances × 3 functions × 3 distances). To evaluate the performance, we use the average modeled probability of speed reduction, $\bar{G}\left(X\right)$ (Equation (3)), as a comparison metric. Statistical significance was assessed at α=0.05 using one-sided t-tests to compare each method against the base case and to compare methods between each other, with p-values adjusted for multiple comparisons.

All experiments were implemented in Python version 3.14, Windows 11. Data visualizations in their real coordinates were carried out using QGIS software version 3.34. The experiments were conducted on an Intel Core Ultra, 14 logical cores and 8 GB of RAM. Each method (NM, GA, and the greedy heuristic) was run 30 times with different random seeds in each of the 18 experimental settings. All three methods (GA, NM, and the greedy heuristic) were initialized from the same hotspot configuration, perturbed uniformly by up to ±250 m in each coordinate. A different perturbation was generated for each run but shared across all three methods to ensure comparable starting conditions. For each setting, we report the mean and standard deviation of $\bar{G}\left(X\right)$ over the 30 runs and the mean runtime per run. Finally, details on the parameters used in each technique are described as follows:

- Nelder–Mead (NM): This algorithm was implemented with the Python library SciPy using the initial positions of the cameras as starting points, and a maximum of 1000 iterations.
- Genetic Algorithm (GA): The GA hyperparameters were calibrated through a preliminary tuning stage using a reduced optimization instance to reduce the computational effort associated with parameter selection. A full factorial search was conducted over the hyperparameter ranges summarized in Table 1, resulting in a total of 81 hyperparameter combinations. Each configuration was evaluated by executing the GA on the reduced optimization instance for three generations. This preliminary stage was intended to rank candidate hyperparameter configurations at a low computational cost rather than to obtain converged solutions; note that, with a fixed number of generations, larger populations involve more objective-function evaluations. The initial population was seeded with the hotspot-based configuration. Table 1 also reports the hyperparameter configuration selected for the final experiments, corresponding to the combination that consistently achieved the best objective function values during the calibration stage. After calibration, the GA was executed on the complete optimization problem using the selected hyperparameter configuration. The algorithm evolved for a maximum of 500 generations and employed an early stopping criterion, terminating the search whenever no improvement in the best objective function value was observed for 50 consecutive generations.
- Greedy heuristic: The greedy relocation heuristic was run for a maximum of 1000 iterations, starting from the perturbed hotspot positions described above, with the movement limit and crash threshold described in Section 4.2.3.

## 6. Results and Discussion

### 6.1. Results

Table 2 presents the average probability of speed reduction, $\bar{G}\left(X\right)$, in Providencia and Santiago Centro for each of the speed reduction functions described in Section 4.1 and each maximum influence distance $d_{\max}$. It covers the base case scenario (the hotspot-based placement) and the three improvement methods: NM, GA, and the greedy heuristic. For each method, the table reports the mean and standard deviation of the final $\bar{G}\left(X\right)$ over 30 independent runs. Table 3 reports the CPU time per run of each method.

The results show that all three methods outperform the base case in all configurations, with the highest adjusted *p*-value being $1.93\times 10^{-27}$. Averaged over the 18 configurations, the improvement was 14.88 pp for GA, 10.65 pp for NM, and 10.48 pp for the greedy heuristic, which correspond to relative improvements of 29.5%, 21.2%, and 20.8%, respectively. The improvements were larger in Santiago than in Providencia (16.15 pp versus 13.60 pp for GA), and the largest improvement, 24.2 pp, was obtained by GA in Santiago with the quadratic function and $d_{\max}=600$ m. Nevertheless, the final solutions remain visually similar to the initial one. This can be attributed to the strong influence of the initial solution on the final one when working with Voronoi diagrams [56].

Under the evaluated budgets and starting configurations, GA achieves the best overall performance, obtaining the highest mean $\bar{G}\left(X\right)$ and the best individual solution in all configurations. In both Santiago and Providencia, GA significantly outperforms NM and the greedy heuristic in all configurations, with the highest adjusted p-value being $1.80\times 10^{-16}$. GA is also the most consistent method, with standard deviations at most 0.31 pp, compared with up to 1.77 pp for NM. The comparison between NM and the greedy heuristic depends on the municipality: NM significantly outperforms the greedy heuristic in all configurations in Providencia (highest adjusted p-value equal to $2.83\times 10^{-6}$), whereas the greedy heuristic outperforms NM in all configurations in Santiago (highest adjusted *p*-value equal to 0.0013).

Finally, the average CPU time per run was 94.5 s for GA, 59.2 s for NM, and 19.1 s for the greedy heuristic (Table 3). Thus, GA provides better solutions at a higher computational cost, requiring about 1.6 times the time of NM and 5 times that of the greedy heuristic. However, in all configurations the average time per run is below 2.5 min on a desktop computer.

### 6.2. Robustness to Roadside Locations

The model allows cameras to be placed anywhere within the study area and measures their influence through Euclidean distance. To assess whether the improvements obtained by the optimization methods depend on these simplifications, we restricted the cameras to feasible roadside locations. For this, the road network was obtained from OpenStreetMap and includes the streets open to motor vehicles (motorway, trunk, primary, secondary, tertiary, unclassified, residential and living street, including their links); service roads and private ways were excluded. Only the parts of the network within the study area and outside the exclusion zones were considered, so that the adjusted configurations satisfy the same constraints as the optimized ones.

Then, each camera of the final configuration of every run, method and setting was moved to the nearest point of this network, with distances measured in meters, and the adjusted locations were transformed back to the normalized study area. The hotspot-based configuration was adjusted in the same way, since the CONASET hotspot coordinates do not coincide exactly with the street centerlines of OpenStreetMap; all configurations are therefore compared on the same road network. $\bar{G}\left(X\right)$ was then re-evaluated for the adjusted configurations with the same model and without further optimization. Table 4 reports $\bar{G}\left(X\right)$ after moving every camera, and every hotspot of the base case, to the nearest street, together with the change with respect to the values before the adjustment. The changes are small: at most 0.35 pp in any configuration, and 0.19 pp or less on average for each method and municipality. As a result, the average gain of GA over the base case changes only from 13.60 to 13.44 pp in Providencia and from 16.15 to 15.98 pp in Santiago, and the ranking of the methods is the same in all configurations: GA obtains the highest value, followed by NM in Providencia and by the greedy heuristic in Santiago. The improvements obtained by the optimization methods are therefore preserved when cameras are restricted to feasible roadside locations.

### 6.3. Coverage of Crash Hotspots

Moving cameras away from the hotspots may leave high-crash sites less protected. To quantify this trade-off, we computed for each hotspot the probability of speed reduction pr(d), where *d* is the distance from the hotspot to the nearest camera of the final solution, and defined the loss as 100%−pr(d)∗100. In the base case, the loss is zero because each hotspot has a camera.

Table 5 highlights the trade-off between maximizing the area-wide probability of speed reduction and preserving coverage at the original crash hotspots. While the optimized layouts improve the overall objective function $\bar{G}\left(X\right)$, relocating cameras away from the hotspots reduces the probability of speed reduction at these locations. Among the three methods, GA consistently exhibits the largest hotspot losses, averaging 32.05 percentage points (pp) in Providencia and 31.24 pp in Santiago, corresponding to retained hotspot coverage probabilities of 67.95% and 68.76%, respectively. NM exhibits intermediate losses of 29.25 pp and 27.41 pp, while the greedy method preserves the highest hotspot coverage, with average losses of 24.85 pp and 25.46 pp, respectively. This pattern is consistent across all experimental configurations. Furthermore, hotspot losses increase as the influence radius *R* decreases, with the logarithmic function producing the largest losses and the quadratic function the smallest. These results reveal an important trade-off: although GA achieves the highest area-wide probability of speed reduction, it does so at the expense of reduced coverage at the original hotspots. Consequently, the proposed optimization framework is particularly advantageous when the objective is to improve speed reduction across the entire study area, rather than prioritizing protection at specific high-risk locations.

### 6.4. Discussion of Results

The results suggest that optimizing camera placement can improve the modeled probability of speed reduction beyond what can be achieved through hotspot-based placement alone. This finding highlights an important distinction between identifying locations with high concentrations of traffic incidents and determining the most effective spatial configuration of cameras. While hotspot-based approaches prioritize locations with high historical risk, optimization methods account for the combined influence of multiple cameras across the study area. Consequently, an optimized configuration may provide greater overall coverage even when individual cameras are not positioned directly at the most prominent hotspots.

**Figure 5:**
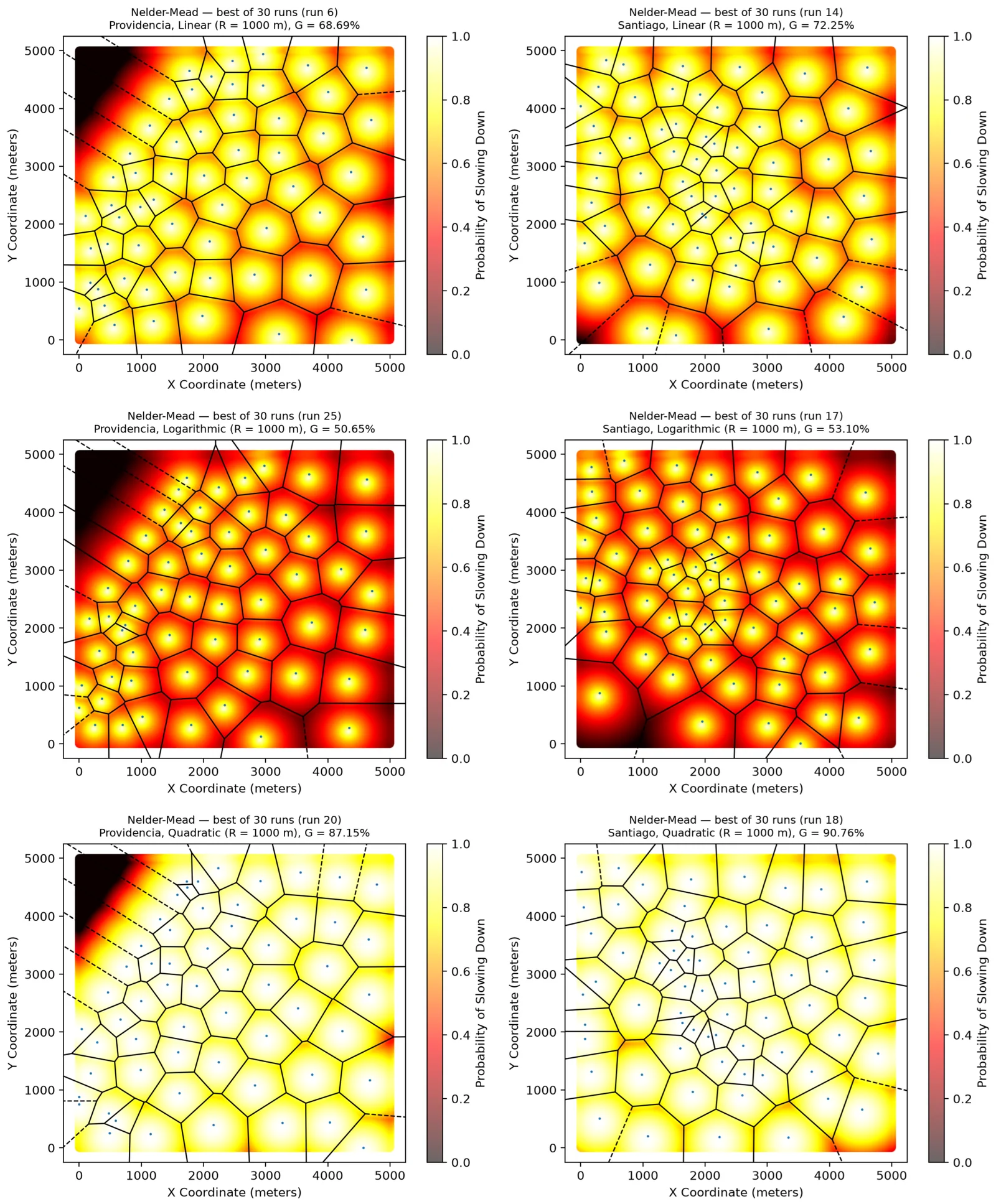
Voronoi Diagrams with the heatmap of the average probability of speed reduction in each Voronoi region using Nelder–Mead algorithm for Providencia (**left**) and Santiago (**right**) for linear (**first row**), logarithmic (**second row**), and quadratic (**third row**) functions, for $d_{max}$ = 1000 m.

**Figure 6:**
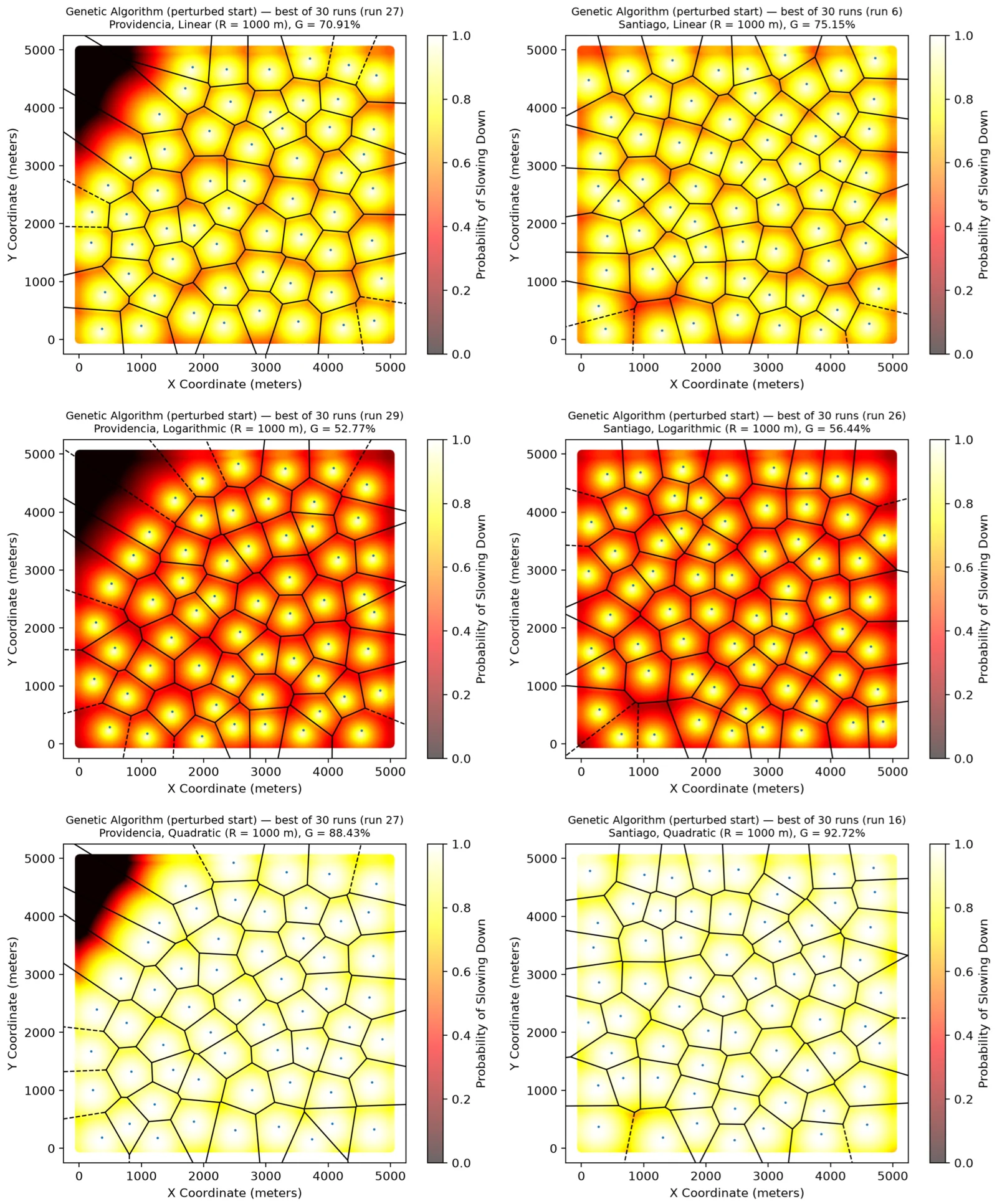
Voronoi Diagrams with the heatmap of the average probability of speed reduction in each Voronoi region using Genetic Algorithm for Providencia (**left**) and Santiago (**right**) for linear (**first row**), logarithmic (**second row**), and quadratic (**third row**) functions, for $d_{max}$ = 1000 m.

The differences observed between Providencia and Santiago further suggest that the potential benefits of optimization depend on the spatial characteristics of the study area and the quality of the initial configuration. The larger improvements in Santiago may indicate greater opportunities to redistribute cameras effectively, whereas the smaller gains in Providencia could suggest that the hotspot-based placement already provides relatively favorable coverage. However, these interpretations require further spatial analysis to establish the underlying mechanisms. As an example of the spatial coverage achieved by the NM and GA algorithms with $d_{max}=1000$, Voronoi diagrams of sample runs are presented in Figure 5 and Figure 6.

The superior overall performance of GA also highlights the importance of the search strategy. Compared with NM and Greedy, GA appears better suited to exploring alternative spatial configurations and identifying improvements that may not be accessible through more localized search procedures.

Another relevant finding is that the benefits of optimization persist across different distance-decay functions and camera influence distances. This suggests that the usefulness of optimizing camera locations is not entirely dependent on a specific representation of how camera influence decreases with distance. However, the magnitude of the improvements varies with these assumptions, emphasizing the importance of sensitivity analysis and appropriate calibration of the distance-decay function. In particular, the assumed influence distance should be interpreted as a modeling parameter rather than an empirically established effective range.

The results also show that restricting the cameras to the road network has only a small effect on the results. The gains over the hotspot-based placement are largely preserved after moving every camera to the nearest street, and the ranking of the methods does not change (Section 6.2). The optimized layouts are therefore robust to the roadside feasibility constraint. This is likely because Santiago and Providencia have dense street networks, so that any point of the study area lies close to a street and the adjustment moves the cameras only a short distance. In less dense areas, such as suburban or rural networks, the adjustment would be larger and a greater loss in coverage could be expected. Testing the method in such areas is left for future work.

One limitation observed from the results is that the optimized layouts increase area-wide coverage at the cost of lower coverage at the crash hotspots (Section 6.3). Whereas the hotspot-based placement has, by construction, no loss at the hotspots, all optimization methods reduce the probability of speed reduction at the hotspots, with the largest loss for GA and the smallest for the greedy heuristic, which only relocates the cameras at the hotspots with the fewest crashes. The loss also depends strongly on the assumed function. It is smallest for the quadratic function, which decays slowly near the camera, and largest for the logarithmic function. These results show a trade-off between area-wide deterrence and protection of known high-risk sites. In that sense, dispersed layouts are preferable when the objective is to reduce speeding over the whole area, whereas hotspot-based placement is preferable when the objective is to protect specific high-crash locations. The optimized layouts should therefore not be interpreted as safer than the hotspot-based placement.

Finally, the improvements reported in this study concern the modeled probability of speed reduction and should not be interpreted as direct evidence of reductions in observed vehicle speeds, crashes, or fatalities. Although empirical evidence links lower average speeds to fewer fatalities [8], establishing the real-world effectiveness of optimized camera placement requires calibrating pr(d) using observed speed data and evaluating the resulting configurations in the field. Future research should also consider additional operational constraints, including installation feasibility, traffic characteristics, and potential behavioral adaptations of drivers, to assess whether the modeled improvements translate into meaningful road safety benefits.

## 7. Conclusions

Studying the location of speed cameras is relevant for reducing speeding and its consequences. Rather than placing speed cameras at crash hotspots, as is usually recommended, this research addresses the problem as a continuous facility location problem in which each camera has an area-wide influence that decreases with distance, approximating the combined halo and spillover effects through an assumed function of the probability of speed reduction. To address this problem, we use Voronoi diagrams to define each camera’s area of influence and develop two algorithms, a Nelder–Mead algorithm (NM) and a Genetic Algorithm (GA), to improve the location of the cameras, comparing them against a greedy relocation heuristic. The probability of speed reduction is modeled through three functions (linear, concave, and convex) and three influence distances (600, 800, and 1000 m).

We tested the methods using crash hotspot data from Santiago and Providencia, Chile [55]. Starting from the hotspot locations, all three methods increased the average modeled probability of speed reduction in all settings, with GA obtaining the best results under the evaluated budgets and starting configurations: an average gain of 14.9 percentage points and up to 24.2 percentage points (Santiago, quadratic function, $d_{\max}=600$ m). The results are robust to the roadside feasibility constraint. That is, moving every camera to the nearest street has only a small effect on the gains and does not change the ranking of the methods. However, the optimized layouts reduce the probability of speed reduction at the crash hotspots, which shows a trade-off between area-wide deterrence and protection of known high-risk sites. Whether the modeled gains translate into lower observed speeds and fewer crashes remains to be verified empirically.

This study has several limitations. First, the speed-reduction functions are assumed rather than estimated, since no empirical study yet relates the probability of speed reduction to the distance from a camera. Calibrating them with observed driver behavior is needed. Second, influence is modeled through Euclidean distance with 360° coverage, which ignores road connectivity, camera orientation, and diversion to untreated roads. Although the results are robust to restricting the cameras to the road network in the two dense urban areas studied, this should be tested in less dense areas, where the adjustment would be larger, and network-based distances would provide a more realistic representation. Third, drivers are assumed to be uniformly distributed, so the objective does not account for differences in traffic volume or crash severity across locations. Future work could weight the objective by mobility demand, combining demand and transport-network information [57]. Fourth, the comparison between methods depends on the computational budgets and starting configurations used. Finally, the number of cameras is fixed. Both methods can be extended to optimize the number of cameras, an extension we are currently developing.

## Figures and Tables

**Table 1:** Genetic algorithm hyperparameter calibration.

Hyperparameter	Candidate Values	Selected Value
Population size	50, 100, 200	200
Crossover rate	0.6, 0.7, 0.8	0.6
Mutation rate	0.001, 0.002, 0.003	0.003
Tournament size	10, 20, 25	20

**Table 2:** Average probability of speed reduction $\bar{G}\left(X\right)$ (%) of the hotspot-based placement and of the final solutions of each method: mean ± standard deviation over 30 independent runs. The best mean of each row is shown in bold.

Function	*R* (m)	Base Case	NM	GA	Greedy
Providencia					
Linear	1000	57.95	67.64 ± 0.86	**70.67 ± 0.13**	66.09 ± 0.55
	800	49.85	60.48 ± 0.99	**64.01 ± 0.19**	58.62 ± 0.57
	600	38.96	49.02 ± 1.00	**53.61 ± 0.25**	46.91 ± 0.66
Logarithmic	1000	41.91	49.69 ± 0.89	**52.53 ± 0.16**	48.15 ± 0.46
	800	35.01	42.74 ± 0.61	**45.70 ± 0.16**	41.02 ± 0.45
	600	26.43	32.82 ± 0.76	**36.14 ± 0.15**	31.31 ± 0.46
Quadratic	1000	75.07	85.96 ± 0.75	**88.21 ± 0.16**	84.45 ± 0.61
	800	66.40	79.59 ± 0.96	**83.16 ± 0.16**	77.89 ± 0.67
	600	53.69	67.36 ± 1.77	**73.62 ± 0.31**	65.42 ± 0.85
Santiago					
Linear	1000	60.26	70.77 ± 0.74	**74.87 ± 0.16**	72.00 ± 0.38
	800	51.69	63.40 ± 0.89	**68.52 ± 0.18**	65.00 ± 0.48
	600	40.06	52.04 ± 1.48	**58.15 ± 0.27**	53.53 ± 0.64
Logarithmic	1000	43.44	52.32 ± 0.66	**56.20 ± 0.14**	53.16 ± 0.39
	800	36.18	45.22 ± 0.79	**49.24 ± 0.15**	46.01 ± 0.42
	600	27.13	35.25 ± 0.95	**39.43 ± 0.16**	35.92 ± 0.48
Quadratic	1000	78.31	89.52 ± 0.56	**92.59 ± 0.11**	90.52 ± 0.27
	800	69.12	83.39 ± 0.92	**88.41 ± 0.14**	85.25 ± 0.44
	600	55.34	71.37 ± 1.53	**79.50 ± 0.30**	74.11 ± 0.73

NM: Nelder–Mead; GA: genetic algorithm; Greedy: greedy movement heuristic. *R*: maximum influence distance of a camera, in meters of the 5000×5000 study area. The three methods start from the hotspots perturbed uniformly by up to ±250 (5% of the side of *S*) in each coordinate, with a different perturbation in each run and the same perturbation for the three methods in a given run; for the GA, the initial population consists of permutations of these perturbed locations. All methods keep the cameras within *S* and outside the exclusion zones.

**Table 3:** CPU time per run (s): mean ± standard deviation over 30 independent runs. All corresponds to the mean over the 540 runs of each method. The calibration of the GA (4374 runs of 3 generations) required an additional 2.8 h of CPU time. Abbreviations as in Table 2.

Function	*R* (m)	NM	GA	Greedy
Providencia				
Linear	1000	52.7 ± 1.2	82.4 ± 14.4	12.7 ± 0.8
	800	51.3 ± 1.1	74.9 ± 14.2	12.9 ± 0.7
	600	49.9 ± 1.2	78.1 ± 15.2	13.7 ± 1.0
Logarithmic	1000	73.5 ± 1.8	107.1 ± 27.7	14.2 ± 0.7
	800	72.9 ± 1.3	108.3 ± 22.7	14.0 ± 0.8
	600	71.0 ± 1.7	108.0 ± 23.1	14.7 ± 1.1
Quadratic	1000	49.0 ± 1.5	75.7 ± 14.9	11.8 ± 0.7
	800	46.7 ± 0.9	70.9 ± 16.0	11.8 ± 0.7
	600	45.2 ± 1.0	67.3 ± 14.1	12.2 ± 0.8
Santiago				
Linear	1000	55.3 ± 1.5	96.5 ± 16.8	24.2 ± 1.6
	800	54.9 ± 1.6	93.4 ± 15.6	24.3 ± 1.6
	600	54.7 ± 2.0	89.4 ± 18.9	24.8 ± 1.6
Logarithmic	1000	79.6 ± 4.7	131.8 ± 27.6	26.9 ± 1.5
	800	78.8 ± 4.3	129.9 ± 25.9	27.2 ± 1.6
	600	76.8 ± 2.6	125.1 ± 24.9	27.7 ± 1.4
Quadratic	1000	52.8 ± 1.5	88.9 ± 16.2	23.2 ± 1.0
	800	51.2 ± 1.3	83.1 ± 15.5	23.5 ± 1.0
	600	49.6 ± 1.2	90.4 ± 12.7	23.7 ± 2.4
All		59.2 ± 12.1	94.5 ± 27.2	19.1 ± 6.3

**Table 4:** Average probability of speed reduction $\bar{G}\left(X\right)$ (%) after moving each camera, and each hotspot of the base case, to the nearest street: mean over 30 independent runs and, in parentheses, mean change with respect to the values before the adjustment (percentage points).

Function	*R* (m)	Base Case	NM	GA	Greedy
Providencia					
Linear	1000	57.98 (+0.03)	67.45 (−0.19)	70.53 (−0.15)	66.11 (+0.02)
	800	49.88 (+0.03)	60.30 (−0.18)	63.87 (−0.15)	58.64 (+0.02)
	600	38.99 (+0.03)	48.77 (−0.25)	53.45 (−0.16)	46.93 (+0.03)
Logarithmic	1000	41.93 (+0.02)	49.57 (−0.12)	52.41 (−0.12)	48.17 (+0.02)
	800	35.03 (+0.02)	42.61 (−0.13)	45.59 (−0.11)	41.05 (+0.03)
	600	26.45 (+0.02)	32.69 (−0.13)	36.03 (−0.11)	31.35 (+0.04)
Quadratic	1000	75.10 (+0.03)	85.74 (−0.22)	88.11 (−0.11)	84.44 (−0.01)
	800	66.44 (+0.04)	79.33 (−0.26)	83.03 (−0.14)	77.88 (0.00)
	600	53.73 (+0.04)	67.10 (−0.26)	73.42 (−0.20)	65.45 (+0.03)
Average		49.50 (+0.03)	59.29 (−0.19)	62.94 (−0.14)	57.78 (+0.02)
Santiago					
Linear	1000	60.24 (−0.02)	70.65 (−0.13)	74.71 (−0.16)	71.96 (−0.04)
	800	51.67 (−0.02)	63.23 (−0.17)	68.35 (−0.18)	64.96 (−0.05)
	600	40.04 (−0.03)	51.87 (−0.17)	57.89 (−0.26)	53.47 (−0.07)
Logarithmic	1000	43.42 (−0.02)	52.22 (−0.10)	56.04 (−0.16)	53.12 (−0.04)
	800	36.15 (−0.02)	45.11 (−0.11)	49.07 (−0.16)	45.97 (−0.04)
	600	27.11 (−0.02)	35.19 (−0.06)	39.26 (−0.17)	35.89 (−0.03)
Quadratic	1000	78.30 (−0.01)	89.37 (−0.15)	92.46 (−0.12)	90.47 (−0.05)
	800	69.10 (−0.01)	83.20 (−0.19)	88.21 (−0.19)	85.18 (−0.07)
	600	55.32 (−0.02)	71.13 (−0.24)	79.15 (−0.35)	74.02 (−0.08)
Average		51.26 (−0.02)	62.44 (−0.14)	67.24 (−0.19)	63.89 (−0.05)

Each camera is moved to the nearest point of the street network open to motor vehicles (OpenStreetMap: motorway, trunk, primary, secondary, tertiary, unclassified, residential, and living street, including links; service roads and private ways excluded), with distances measured in real meters, and G(X) is recomputed with the same model. The hotspots of the base case are adjusted in the same way, so that all configurations are compared on the same street network. The adjusted positions are kept within the study area and outside the exclusion zones.

**Table 5:** Average loss of probability of speed reduction at the hotspots (%): mean ± standard deviation over 30 independent runs. For each hotspot, the loss is 100%−pr(d)∗100, where *d* is the distance from the hotspot to the nearest camera of the final solution.

Function	*R* (m)	NM	GA	Greedy
Providencia				
Linear	1000	23.15 ± 1.64	24.73 ± 1.36	18.83 ± 1.18
	800	28.26 ± 1.97	30.94 ± 1.65	23.54 ± 1.51
	600	36.56 ± 2.10	41.03 ± 2.40	31.20 ± 1.81
Logarithmic	1000	39.78 ± 1.86	43.31 ± 1.36	35.35 ± 1.65
	800	46.12 ± 1.95	50.55 ± 1.31	41.28 ± 1.89
	600	55.00 ± 2.43	59.49 ± 2.26	49.81 ± 2.19
Quadratic	1000	6.72 ± 0.78	7.29 ± 0.64	4.48 ± 0.49
	800	10.59 ± 1.19	11.28 ± 1.15	6.98 ± 0.83
	600	17.08 ± 1.67	19.85 ± 2.04	12.19 ± 1.33
Average		29.25	32.05	24.85
Santiago				
Linear	1000	20.98 ± 1.54	24.43 ± 1.46	19.29 ± 1.17
	800	25.80 ± 1.91	29.22 ± 1.83	24.07 ± 1.47
	600	34.23 ± 2.09	39.67 ± 2.18	32.07 ± 1.96
Logarithmic	1000	38.08 ± 1.97	42.58 ± 1.59	35.86 ± 1.65
	800	44.52 ± 1.78	49.66 ± 2.64	41.91 ± 1.95
	600	53.29 ± 2.40	59.46 ± 2.10	50.66 ± 2.23
Quadratic	1000	6.04 ± 0.82	6.88 ± 0.78	4.73 ± 0.44
	800	8.94 ± 1.42	10.42 ± 0.98	7.42 ± 0.76
	600	14.81 ± 2.18	18.88 ± 1.72	13.14 ± 1.43
Average		27.41	31.24	25.46

## Data Availability

Data are available upon request.
